# ERR and dPECR Suggest a Link Between Neuroprotection and the Regulation of Ethanol Consumption Preference

**DOI:** 10.3389/fpsyt.2021.655816

**Published:** 2021-04-26

**Authors:** Laura Velo Escarcena, Margarita Neufeld, Marcella Rietschel, Rainer Spanagel, Henrike Scholz

**Affiliations:** ^1^Institute of Zoology, University of Köln, Köln, Germany; ^2^Department of Genetic Epidemiology in Psychiatry, Central Institute of Mental Health (CIMH), Heidelberg University, Mannheim, Germany; ^3^Institute of Psychopharmacology, CIMH, Heidelberg University, Mannheim, Germany

**Keywords:** hangover, ERR, PECR, neuroprotection, ethanol consumption preference, *Drosophila*

## Abstract

Reconsumption of ethanol after withdrawal is a hallmark for relapse in recovering patients with alcohol use disorders. We show that the preference of *Drosophila melanogaster* to reconsume ethanol after abstinence shares mechanistic similarities to human behavior by feeding the antirelapse drug acamprosate to flies and reducing the ethanol consumption preference. The *Drosophila* cellular stress mutant *hangover* also reduced ethanol consumption preference. Together with the observation that an increasing number of candidate genes identified in a genome-wide association study on alcohol use disorders are involved in the regulation of cellular stress, the results suggest that cellular stress mechanisms might regulate the level of ethanol reconsumption after abstinence. To address this, we analyzed mutants of candidate genes involved in the regulation of cellular stress for their ethanol consumption level after abstinence and cellular stress response to free radicals. Since *hangover* encodes a nuclear RNA-binding protein that regulates transcript levels, we analyzed the interactions of candidate genes on transcript and protein level. The behavioral analysis of the mutants, the analysis of transcript levels, and protein interactions suggested that at least two mechanisms regulate ethanol consumption preference after abstinence—a nuclear estrogen-related receptor-hangover-dependent complex and peroxisomal trans-2-enoyl-CoA reductase (dPECR)-dependent component in peroxisomes. The loss of estrogen-like receptor and dPECR in neurons share a protective function against oxidative stress, suggesting that the neuroprotective function of genes might be a predictor for genes involved in the regulation of ethanol reconsumption after abstinence.

## Introduction

Alcohol is one of the most commonly used drugs worldwide. Repetitive consumption and abusive use can result in alcohol use disorders (AUDs), and a genetic predisposition contributes 40–60% to the development of AUDs (1, 2). To identify genetic variants correlating with a high risk of developing AUDs, genome-wide association studies (GWAS) have been performed with AUD patients. Several single-nucleotide polymorphisms (SNPs) have been associated with AUDs (3–5). Whether the SNPs are associated with genes involved in the development of AUDs or with behaviors associated with AUDs is mostly unknown.

Genetic model systems have been successfully employed to validate the function of candidate genes implicated in AUDs and to discover novel molecular players in AUD-related behaviors (6–8). For example, a GWAS showed that X-ray repair complementing defective repair in Chinese hamster cells 5 (XRCC5) was associated with AUDs. Follow-up studies with *Drosophila melanogaster* and humans confirmed a role for XRCC5 as a risk gene for alcohol dependence (8). The genetic model organism *Drosophila melanogaster* shares behavioral similarities with humans, such as the development of ethanol tolerance and the preference to consume ethanol after deprivation, as well as mechanistic similarities (9). For example, phenotypic analysis of *Drosophila hangover* mutants (*hang*) revealed a cellular stress pathway that functions within neurons and is required for ethanol tolerance (10). Polymorphisms in the *hang*-related gene ZNF699 are associated with AUDs in a population of Irish patients (11).

Here, we addressed whether the preference to consume ethanol in *Drosophila melanogaster* is mechanistically similar to relapse after withdrawal in humans using pharmacology. Next, we analyzed whether we can identify mutants with specific changes of ethanol consumption preference after abstinence by analyzing the ethanol reconsumption behavior of *hang* mutants. To identify new regulators of ethanol reconsumption behavior, we analyzed the function of selected candidate genes identified with AUDs based on a GWAS (5, 8) in ethanol reconsumption preference after abstinence. *hang* encodes a nuclear RNA-binding protein and regulates transcript level (12). Thus, transcripts regulated by Hang might also regulate ethanol reconsumption preference after a period of abstinence. To identify cellular components that regulate the preference to consume ethanol after abstinence, we investigated interaction of *hang* and candidate genes on transcript and protein level. Finally, we analyzed the neuronal function of candidates in regulation of cellular stress to free radicals. Our approach revealed new mechanisms regulating the level of ethanol intake after deprivation and validated GWAS-identified candidate genes for AUD-related behavior.

## Materials and Methods

Flies were raised under standard conditions (13). For fly stock, see Supplementary Table 1. Flies carrying transposable element insertions marked with *white* were backcrossed to *w*^1118^ (Scholz lab) for at least *five* generations to isogenize the genetic background. 1–3-day-old adult male flies were collected under CO_2_ sedation, and 2 days later, used for experiments.

Alcohol intake and preference was measured using a capillary feeder (CAFÉ) (13). A group of eight 3-day-old male flies were given the choice between two capillaries filled with 5% sucrose and two capillaries filled with 15% ethanol containing 5% sucrose. To control for evaporation, at least three assays without flies were used to determine the average evaporation rate per day. Consumption preference of ethanol after abstinence assay was measured after (14). For pharmacological experiments, a concentration of 0.024 μg/μL acamprosate was added to all solutions.

To test for oxidative sensitivity, ten 3–4-day-old male flies were placed in a medium culture vial containing filter papers soaked with 800, μL of a 5% H_2_O_2_ containing 5% sucrose solution, and survival was monitored every 12 h similar as described in Bayliak et al. (15).

Transcripts were measured by quantitative real-time PCR (qRT-PCR). Flies were exposed to ethanol vapor until 50% lost their righting reflex. After 4 h of recovery on normal food RNA was isolated. Transcript levels were compared with transcript levels of flies that underwent a mock treatment. In brief, total RNA was isolated from 20 fly heads using TRIzol®. RNA was resuspended in 50 μL of ddH_2_O and digested with RNase-free DNAse for 1 h at 37°C followed by heat inactivation for 10 min at 95°C. cDNA was synthesized with SuperScript™II Reverse Transcriptase (Invitrogen™) and oligo dT primers. One hundred nanograms of cDNA were used as the template for qRT-PCR analysis with SYBR® master mix (Eurogentec). The experiments were carried out using an iCycler iQ5 Multicolour Real-Time PCR Detection System and its corresponding iQ5 Optical System Software from Bio-Rad. The data were analyzed using the ΔΔCt method (16). Primers spanning introns of the candidate genes were used, and control genes were identified using NormFinder software (17) (Supplementary Table 2 shows the list of primers). cDNA was isolated from at least four different sets of flies, and qRT-PCR was performed with three technical replicates. The data are presented as fold changes of the candidate gene transcripts relative to the control gene transcripts.

Significance was determined using Student's *t* test for two groups and for more than two groups one-way analysis of variance (ANOVA) followed by *post hoc* Tukey-Kramer adjustment. For multiple comparisons, the significance level was adjusted with Bonferroni correction. All graphs represent mean ± s.e.m. Unless otherwise specified, *n* refers to the number of tested groups of flies. Statistica Software Version 9 was used for statistical analysis.

## Results

Given the choice, flies prefer to consume ethanol-enriched food to non-alcohol-containing food (18). This might be due to the intoxicating effect of ethanol (14). After abstinence, they resume to prefer feeding on ethanol-enriched food similar to what is observed in humans after relapse (14).

### Acamprosate Reduces Ethanol Consumption Preference in Flies

**Figure 1:**
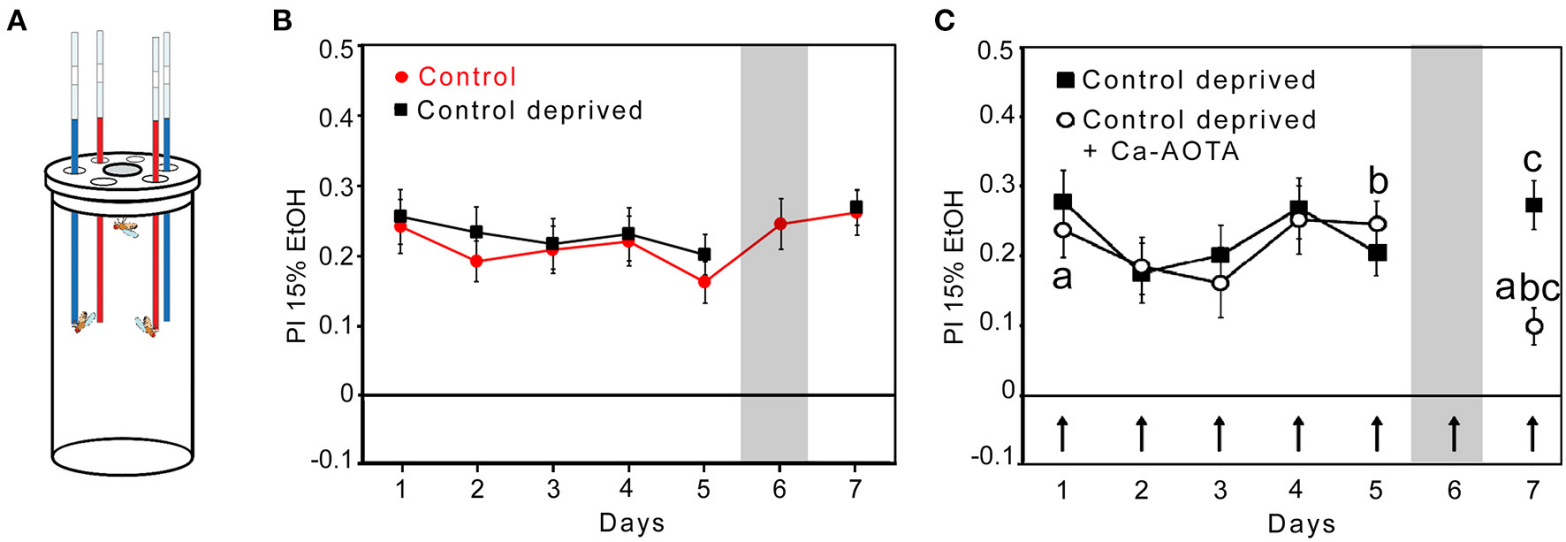
Mechanistic similarities between ethanol consumption preference in flies and relapse in humans. **(A)** Capillaries are filled with 15% EtOH containing 5% sucrose (red) and 5% sucrose (blue). The preference to consume ethanol-containing food was defined by the relative difference between ethanol-enriched and non-ethanol-enriched food in comparison with the total amount of food consumed. **(B)** Control flies significantly preferred to consume ethanol-enriched food for 7 days. Ethanol-deprived flies on day 6 showed ethanol consumption preference again on day 7. *N* = 15 groups after deprivation. **(C)** Acamprosate (Ca-AOTA) mixed into all solutions of the experimental groups is indicated by an arrow. After deprivation, the flies showed significantly reduced ethanol consumption preference compared with the controls at day 7 (indicated by the letter “c”; *P* < 0.001), at day 5 prior deprivation (indicated by the letter “b”; *P* < 0.01), and the 1st day (indicated by the letter “a; *P* < 0.01). *N* = 28. Significance was determined using a two-tailed *t* test, and differences from random choice were determined using a one-sample sign test. The data are presented as the means ± s.e.m.

To test whether the preference to consume ethanol in flies underlies similar pharmacological properties as relapse behavior in humans, we tested whether acamprosate, a clinically approved medication for alcohol relapse prevention (19), is able to reduce ethanol consumption preference after abstinence in flies. To measure voluntary ethanol intake, we used a capillary feeder assay (Figure 1A). Flies preferred to feed on 15% ethanol enriched with 5% sucrose for five consecutive days (Figure 1B). On day 6, one-half of the flies continued to have a choice between different sucrose and sucrose + ethanol, whereas the experimental groups had access only to 5% sucrose for 24 h. After abstinence, and the re-introduction of ethanol, the experimental group significantly preferred to consume ethanol. They did not differ in their preference to controls that did not undergo abstinence (Figure 1B). Since in humans, steady-state concentrations of acamprosate is reached only after 5 to 7, days (20), Acamprosate was added to both solutions from days 1 to 7 (Figure 1C). During the first 5, days, the acamprosate-treated flies had similar levels of fluid intake (controls: 3.59, ±, 0.14, μL per fly and the experimental flies: 3.63, ±, 0.13, μL per fly). After abstinence, the acamprosate-treated flies significantly decreased their ethanol preference compared with that of the control flies and their own baseline ethanol consumption preference on days 1 and 5 predeprivation (Figure 1C). Acamprosate reduced preference to consume ethanol-containing food, similar to what is seen in comparable animal models and humans. Thus, these data provide pharmacological validity to the *Drosophila* model of alcohol relapse.

### Reduced Ethanol Consumption Preference After Abstinence in *Hang, dPECR*, and *ERR* Mutants

**Figure 2:**
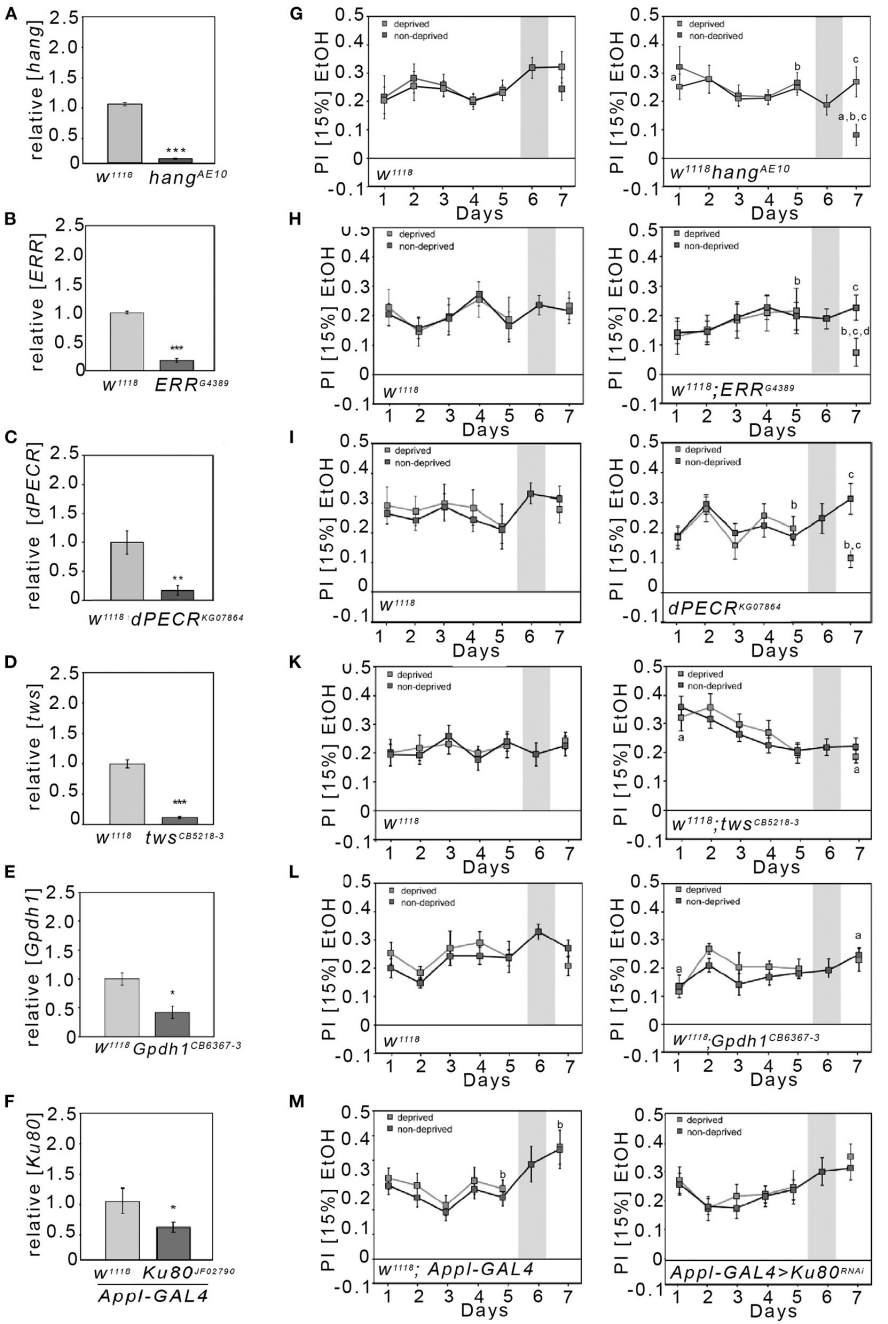
Reduction in the preference to consume EtOH after abstinence in *hang, ERR*, and *dPECR* mutants. **(A–F)** The qRT-PCR analysis performed on *polyA*-selected head RNA revealed a significant reduction in transcripts of candidate genes (transcript levels for *hang*^*AE*10^: 0.08 ± 0.01; for *ERR*^*G*4389^: 0.17 ± 0.02; *dPECR*^*KG*07864^: 0.17 ± 0.04; *twins*^*CB*5218−3^: 0.11 ± 0.02; *Gpdh1*^*CB*6367−3^: 0.42 ± 0.11; for transheterozygous flies carrying a *Ku80* RNAi transgene under the control of the *Appl*-Gal4 driver: 0.55 ± 0.09). *N* = 4 different RNA samples; data are presented as the means ± STDEV. Significance was determined using a two-tailed Student's *t* test. ^*^P < 0.05, ^**^P < 0.01, and ^***^P < 0.001. **(G–M)** Two different groups of flies were tested—the respective control for the mutants (**G**–**M** first panel) and the mutants (**G**–**M** second panel). The control group and mutant groups were further divided into two groups. The control group continuously had access to both solutions from days 1 to 7, whereas the experimental group on day 6 had only access to food without ethanol and on day 7 access to both solutions again. **(M)** Heterozygous *Appl*-Gal4 flies were compared with the transheterozygous flies, which were carrying one copy of the RNAi transgene and one copy of the *Appl*-Gal4 transgene. **(G–M)** The letter “a” indicates a significant difference between the ethanol consumption preference on days 1 and 7; the letter “b” indicates a difference between day 5 prior to the deprivation of ethanol and day 7; and the letter “c” indicates the significant difference between the control group and deprived group at day 7. For a–c, *P* < 0.016. *N* = 8–17. The data are presented as the means ± s.e.m. Significance was determined using a two-tailed *t* tests. To determine whether flies preferred ethanol and did not choose at random between the two offered solutions, the one-sample sign tests were used.

To analyze whether we can identify mutants with selective defects in their ethanol consumption preference after abstinence, we analyzed *hang* mutants for their preference to consume ethanol-enriched food over non-ethanol-containing food after abstinence (Figure 2). The *hang* gene defines a major cellular stress pathway underlying behavioral responses to repetitive ethanol exposure (10). During the initial 5 days, *hang*^*AE*10^ flies consistently preferred ethanol-containing food. After deprivation, on day 7, the deprived group significantly reduced their ethanol intake in comparison with *hang* mutants that did not undergo a period of abstinence. The preference of the deprived *hang* flies was also significantly lower than the preference at the start of the experiment and before deprivation. Thus, *hang* mutants show a selective defect in ethanol preference after abstinence.

Next, we wanted to identify novel players underlying the regulation of ethanol preference after abstinence. We thought to identify these genes in a set of preselected gene loci implicated in alcohol use disorder by GWAS. As entry point, we focused our analysis on the GWAS performed by Treutlein et al. (5). First, we selected candidate genes that had been tagged by SNPs identified with AUDs by GWAS and in follow-up studies [see Table 1 in Treutlein et al. (5)]. The list consists of 16 SNPs; 12 of which are localized within introns (one SNP is associated with the introns of two genes and two additional SNPs are inserted in the intron of one of the genes without affecting the other) and four are between genes. In total, the SNPs are associated with 19 different genes (Supplementary Table 3). Two genes that encode a nuclear RNA and a pseudogene were excluded from the analysis. For the remaining 17 human candidate proteins, we performed a BLASTP analysis with the *Drosophila melanogaster* proteome. We further excluded calpastatin, interferon regulatory factor 5 (IRF5), and centrosomal protein 83 (CEP83) because we did not detect any similar proteins in *Drosophila*. In our analysis, we also included the Ku80, the *Drosophila* homolog of human XRCC5, because previous studies implicated Ku80 in the susceptibility to AUDs and because previous studies implicated Ku80 in cellular stress responses (8, 21).

Since four out of the remaining 14 genes are known to be involved in the regulation of cellular stress (Supplementary Table 3) as well as the *hang* (10) and *Ku80* (21), we focused our analysis on the following candidate genes: peroxisomal trans-2-enoyl-CoA reductase (PECR), serine/threonine-protein phosphatase 2A, 55 kDa regulatory subunit B beta isoform (PPP2R2B), estrogen receptor 1 (ESR1), glycerol-3-phosphate dehydrogenase 1-like (GPD1L), and XRCC5. The *Drosophila* CG10672/dehydrogenase/reductase 4 protein is with 35% amino acid identity the most similar to human PECR; thus, we named this candidate gene from here on *dPECR*. The *Drosophila* Twins is the most similar to the human PPP2R2B protein, with 79% amino acid identity. The most similar protein to the human ESR1 protein in *Drosophila* is the estrogen-related receptor (ERR), with 35% amino acid identity. Human glycerol-3-phosphate dehydrogenase 1-like (GPD1L) shares the highest homology, with approximately 60% identity, to *Drosophila* glycerol-3-phosphate dehydrogenase 1 (Gpdh1).

First, we screened for viable homozygote mutants. Next we determined the level of transcripts used qRT-PCR with cDNA isolated from adult *Drosophila* heads (Figure 2). For five mutant lines, homozygous flies were viable and qRT-PCR analysis performed on polyA-selected head RNA revealed a significant and pronounced reduction in transcripts of the respective candidate genes (Figures 2A–E). However, no viable mutants for *Ku80* were available. Therefore, we used RNA interference (RNAi) to reduce *Ku80* expression under the control of *Appl*-Gal4 in a broad set of neurons (Figure 2F). Next, we analyzed the mutant flies to determine their baseline ethanol preference and ethanol reconsumption after deprivation. The control for the respective mutants consumed ethanol after a period without ethanol to a similar extend as flies that did not undergo a period without ethanol (Figures 2G–M). In contrast to the controls, the *dPECR* and *ERR* mutant flies showed significantly reduced preference for ethanol after deprivation (Figures 2H,I). The reduction in *twins, Gpdh1*, or neuronal *Ku80* did not alter ethanol reconsumption after abstinence (Figures 2K–M). Whether these candidate genes can be completely excluded from playing a role in regulating ethanol preference after abstinence remains to be determined, since *twins* mutation showed significantly increased ethanol preference on day 1 compared with day 7 after abstinence, and the knockdown of *Ku80* and *Gpdh1* might not have been sufficient to alter gene function to influence ethanol consumption preference.

In summary the *hang, dPECR*, and *ERR* mutant flies were specifically impaired in terms of ethanol consumption after abstinence and together with the observation that acamprosate reduces ethanol consumption reference these results suggest that *dPECR, ERR*, and *hang* regulate relapse-like behavior.

### Neuroprotection Against Oxidative Stress Due to the Loss of *dPECR* and *ERR*

**Figure 3:**
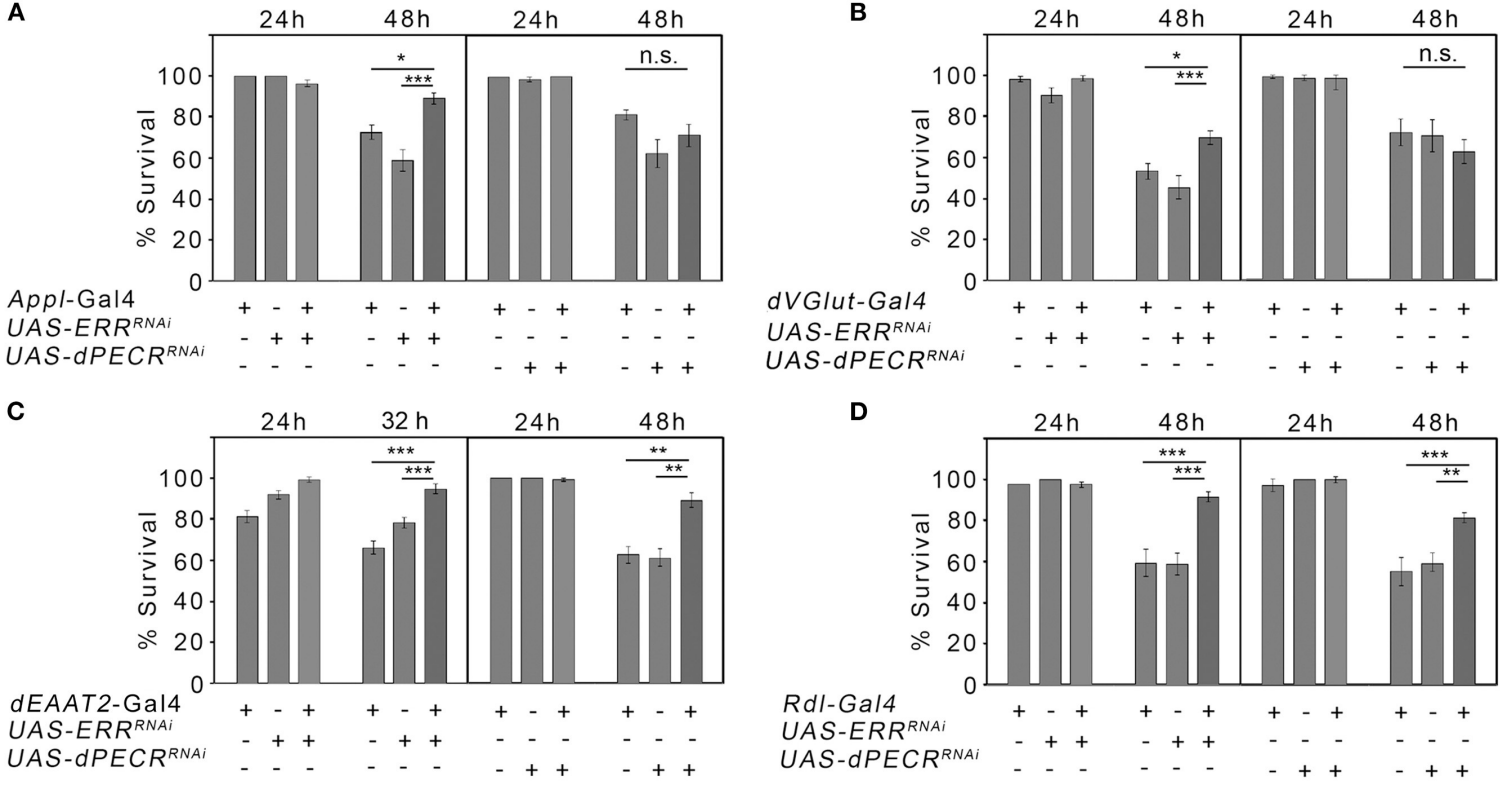
Neuroprotection against oxidative stress due to the loss of ERR and dPECR. **(A–D)** Neuron-type-specific knockdown of *ERR* and *dPECR* under the control of different Gal4 drivers revealed significant differences in stress resistance against the oxidative stress of the controls and the experimental groups. *N* = 16–33 groups of 10 flies. The data are the means ± s.e.m. Significance was determined using one-way analysis of variance followed by *post hoc* Tukey-Kramer adjustment. *P* < 0.05, ^**^P < 0.01, and ^***^P < 0.001.

Since the here-studied genes are known to be involved in the regulation of cellular stress [Supplementary Table 3; (22, 23)], we next asked whether changes in stress sensitivity in neurons correlates with changes in ethanol reconsumption after abstinence. Ethanol promotes the formation of free radicals that cause cellular stress and in the end cell death that might affect the survival of the organism (24). Therefore, we analyzed whether the reduction in *ERR* and *dPECR* function in neurons changes the survival rate in response to the free radical forming agent H_2_O_2_. We reduced *ERR* and *dPECR* using RNAi transgenes in different sets of neurons using different GAL4 drivers and assayed survival in response to oxidative stress (Figure 3). First, to insure that the transgenes are functional, we determined that the expression of the RNAi transgenes *UAS-ERR*^*miRNA*^ and *dPECR*^*HMS*00752^ under the control of the *Appl*-Gal4 driver effectively reduced gene expression using qRT-PCR (Supplementary Figure 1). For the survival experiments, the function of ERR and dPECR were reduced in a broad set of neurons using the *Appl*-Gal4 driver (25). Different types of neurons are differently sensitive to cellular stress. To alter gene function selectively in glutamatergic, taurine-containing neurons, or GABAa receptor-expressing neurons, we used the following Gal4 drivers: the *DVGlut*-Gal4 driver to target glutamatergic neurons that express the vesicular glutamate transporter (26); the *dEAAT2*-Gal4 driver to target neurons that express the excitatory amino acid transporter 2 (dEAAT2) and to label taurine-containing neurons (27); and the *Rdl*-Gal4 driver to target neurons that express the GABA_A_ receptor resistance to dieldrin (Rdl), a postsynaptic marker for GABAergic signaling (28). Reduction in *ERR* in a broad set of glutamatergic, dEAAT2-expressing and Rdl-expressing neurons significantly increased resistance to stress caused by oxygen radicals (Figures 3A–D). The reduction in *dPECR* in a broad set of neurons or glutamatergic neurons did not interfere with survival, but reduction in *dPECR* in taurine-containing neurons and Rdl-expressing neurons significantly increased resistance to free radicals (Figures 3A–D). Thus, loss of *ERR* and *dPECR* protects neurons against oxidative stress and *dPECR* especially in specific subset of neurons.

### Ethanol Affects *ERR* and *dPECR* Differently

**Figure 4:**
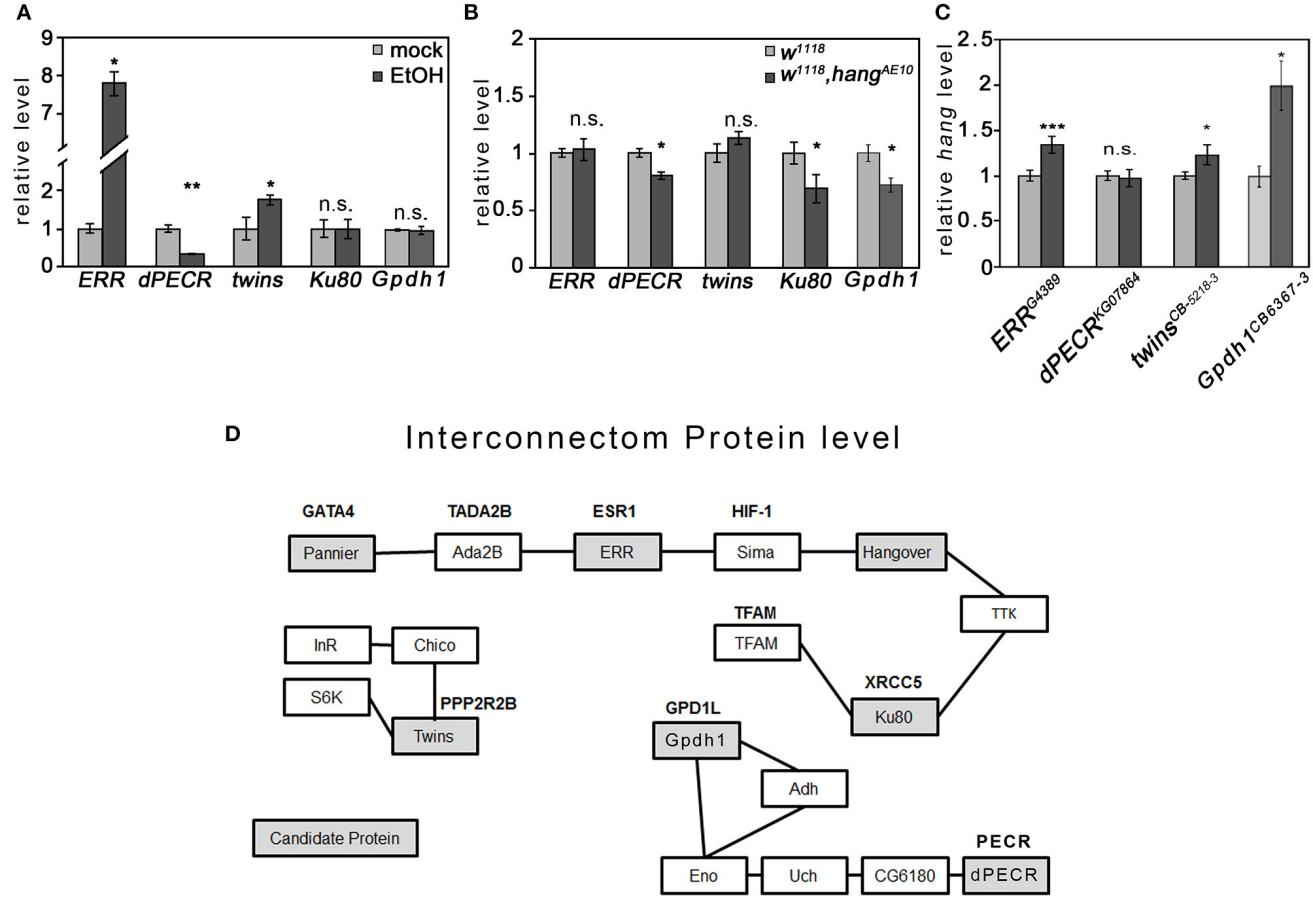
Interaction of candidate genes and proteins. **(A)** Ethanol significantly increased the *ERR* and *twins* transcript levels and significantly reduced the *CG10672/dPECR* transcript level in the fly heads. **(B)** In ethanol-naïve *hang*^*AE*10^ mutant flies, the *CG10672/dPECR, Ku80*, and *Gpdh1* transcript levels were significantly reduced. **(C)** In ethanol-naïve *ERR, twins*, and *Gpdh1* mutants, the *hang* transcripts were significantly reduced. *N* = 4 different RNA samples; the data are presented as the means ± STDEV. Significance was determined using a two-tailed *t* test. ^*^P < 0.05, ^**^P < 0.01, and ^***^P < 0.001. **(D)** The interconnection analysis at the protein level was generated by a data bank analysis and was supported by experimental data as described in Supplementary Table 4.

Changes in transcript levels in response to ethanol are widely used as markers for genes involved in the regulation of ethanol-induced behaviors. Thus, we determined whether the transcript levels of the selected candidate genes changed upon ethanol exposure in fly heads using qRT-PCR analysis (Figure 4). We focus on difference in the head, since we were interested in detecting difference in genes that might regulate behavior. After the ethanol vapor exposure 4 h later, the transcript level of *ERR* was significantly increased by approximately 8-fold and the transcript level of *twins* by approximately 1.7-fold, whereas the transcript level of *dPECR* was significantly reduced by approximately 68% (Figure 4A).

The *hang* gene encodes a neuronal nuclear RNA-binding protein that regulates transcript levels (12), and the *ERR* encodes a nuclear hormone receptor that induces a transcriptional switch (29). To investigate whether *hang* regulates the transcript of candidate genes, the transcript levels of the candidate genes were determined in the heads of the *hang*^*AE*10^ mutants (Figure 4B). The transcripts of *ERR* and *twins* were not changed, but those of *dPECR, Gpdh1*, and *Ku80* were significantly reduced. To investigate whether these genes regulate *hang*, the *hang* transcript level was analyzed in the heads of the respective mutant flies (Figure 4C). In *ERR, Gpdh1*, and *twins* mutants, *hang* was significantly upregulated, whereas in the *dPECR* mutants, *hang* was not changed. In summary, ethanol increased the transcript levels of *ERR* and *twins*, and both negatively regulated the transcript level of *hang*; whereas, ethanol suppressed the *dPECR* transcript level, and *hang* is a positive regulator for *dPECR, Ku80*, and *Gpdh1* transcript levels. Thus, *ERR* is ethanol inducible and upstream of the *hang* transcription or RNA stability or RNA turnover, whereas *hang* is upstream of *dPECR* and is a positive regulator of *dPECR*.

To identify physical interactions of the candidate proteins, we performed a database analysis to identify protein-protein interactions (Figure 4D; Supplementary Table 4). We identified three different groups of interactions at the protein level. The first group contained nuclear proteins such as ERR, Ku80, pannier, and Hang, for which we found indirect protein-protein interactions *via* one additional shared partner, Sima.

For certain shared partners, genetic and protein interactions have been previously shown (Supplementary Table 4). For example, Ku80 together with Sima/Hif-1α regulates the PDK-1 function required for apoptosis (30), and ERR binds to Sima/Hif-1α to induce the Hif-1α-dependent transcriptional program under hypoxia (23), whereas the interaction of Sima and Hang was identified by coimmunoprecipitation (31). Whether these interactions happen at the same time needs to be further analyzed. Twins belongs to the second group of proteins, which are involved in insulin signaling. The final group contained dPECR and Gpdh1. dPECR interacts with several other proteins before interconnecting with Gpdh1, indicating that dPECR and Gpdh1 have a very indirect interaction. dPECR and ERR were not found in the same interacting protein-protein complex, which is not surprising given the different subcellular locations of these proteins.

## Discussion

Acamprosate reduced the ethanol consumption preference of flies, similar to the reduced relapse behavior after withdrawal observed in humans and rats (32, 33). Thus, mechanistic similarities can be used to reveal mechanisms regulating the preference to consume ethanol. We show that in flies, genes involved in the regulation of cellular stress responses are also involved in the regulation of ethanol consumption preference after abstinence. The *hang* gene is required for ethanol reconsumption and the regulation of cellular stress (10). The *dPECR* and *ERR* mutants reduced the ethanol preference of the flies after abstinence, and losses of *dPECR* and *ERR* conferred neuroprotection against oxidative stress. PECR is required for fatty acid biosynthesis in peroxisomes (22). Fatty acid synthesis and degradation regulate oxidative stress responses in cancer cells (34). Loss of dPECR, specifically in taurine-containing neurons and Rdl-expressing neurons, is neuroprotective. Hypoxia increases taurine release (35), and taurine confers neuroprotection against oxidative stress (36). Acamprosate as a calcium acetyl homotaurine is neuroprotective and inhibits the binding of taurine to taurine receptors, thereby altering taurine signaling (37, 38). In addition, acamprosate decreases brain lipid peroxidation and the activity of brain antioxidant vitamins (39). ERR together with HIF-1α directly regulates the HIF-1α-dependent transcriptional program in response to hypoxia (23). Thus, our results suggest a link between dPECR- and ERR-induced neuroprotection and the regulation of ethanol preference after abstinence.

At least two mechanisms regulate the preference to consume ethanol after abstinence. The first mechanism is ERR dependent and regulates changes in transcript levels. Ethanol increases the expression of *ERR* in the fly brain. Similar to the expression of ERR in fly heads, in mice, *ESR1* is also expressed in the brain (40), and in rats, long-term exposure to ethanol increases *ESR1* expression in the brain (41). ERR is a nuclear receptor that interacts with the transcription factor Sima/Hif-1α to regulate transcription (23). Here, we show that ERR is a negative regulator of *hang*. In addition, ERR and Hang bind to each other at the protein level *via* Sima/Hif-1α (23, 31). The Hang protein is expressed in the nucleus of neurons and regulates the transcript levels of genes such as *dunce, dPECR, Ku80*, and *Gpdh1* (10, 12). The localization of Hang and ERR in the nucleus and the transcriptional changes observed suggest that the regulation of ethanol preference after abstinence requires transcriptional changes. The mechanism might be conserved between flies and vertebrates, since in female rats, blockade of ESR1 tended to show reduced reestablishment after withdrawal (42), and the lack of ESR1 in the ventral tegmental area reduces binge-like ethanol drinking in female mice (43). In human male patients, an increase in serum estradiol—the ligand for estrogen receptors—during withdrawal correlated with a higher likelihood of more frequent and earlier alcohol-related hospital readmissions, suggesting that estrogen signaling influences the rate of relapse (44).

The second mechanism depends on dPECR function in peroxisomes. *dPECR* is expressed in fly heads (45) (Figure 2) and is required for the elongation of fatty acid chains (22, 46). In *Drosophila* larvae, exposure to ethanol disrupts fatty acid metabolism (47). Consistent with the disruption of fatty acid metabolism due to ethanol exposure, we found a reduction in *dPECR* after ethanol exposure. PECR requires NADPH to function (22), as well as the catalase expressed in peroxisomes and the major enzyme for ethanol metabolism in the brain (48, 49). The requirement of NADPH links PECR to catalase function and ethanol metabolism.

In conclusion, this work suggests that the ERR-Hang complex and the dPECR function link the regulation of transcript level and the function of peroxisomes to changes in neuronal plasticity and neuroprotection against oxidative stress. The similarity of ERR and dPECR to the human ESR1 and PECR suggests that these results could be transferred to higher organisms.

## Data Availability Statement

The original contributions generated for this study are included in the article/Supplementary Material, further inquiries can be directed to the corresponding author/s.

## Author Contributions

LV designed the work and acquired and analyzed the data. HS designed the work, analyzed the data, and together with RS wrote the manuscript. MN acquired data. MR and RS designed the work. RS provided acamprosate. All authors contributed to the article and approved the submitted version.

## Conflict of Interest

The authors declare that the research was conducted in the absence of any commercial or financial relationships that could be construed as a potential conflict of interest.
