# Supplementary material for: ERR and dPECR Suggest a Link Between Neuroprotection and the Regulation of Ethanol Consumption Preference
Source: Front Psychiatry. 2021 Apr 26;12:655816. doi: 10.3389/fpsyt.2021.655816 (PMC8107284; doi:10.3389/fpsyt.2021.655816)
Supplement: Supplementary file 1 [file Data_Sheet_1.PDF]

ERR and dPECR suggest a link between neuroprotection and the regulation of ethanol consumption preference

Laura Velo Escarcena[1], Margarita Neufeld[1], Marcella Rietschel[2], Rainer Spanagel[4], Henrike Scholz[1]*

[1]Institute of Zoology, University of Köln, Zülpicher Straße 47B, 50674 Köln, Germany

[2]Department of Genetic Epidemiology in Psychiatry, Central Institute of Mental Health (CIMH), Mannheim

[3]Department of Addictive behaviour and Addiction Medicine, CIMH, Mannheim

[4]Department of Psychopharmacology, CIMH, Mannheim

SUPPLEMENTAL INFORMATION contains four tables and one figure.

Supplementary Table 1. Fly Stocks.

Name	Genotype	Gene	Origin
w^{1118}	*w*^{1118}	*W*	Scholz lab, genetic background
hang^{AE10}	*w*^{1118}, *hang*^{AE10}	*Hang*	Scholz et al., 2005
Appl-Gal4	*w*^{1118}*;;Appl-Gal4*	*Appl*	Torroja et al., 1999
dEAAT2-Gal4	*w*^{1118}*;dEAAT2-Gal4*	*dEAAT2*	Besson et al., 2011
Rdl-Gal4	*w*^{1118}*;Rdl-Gal4*	*Rdl*	Kolodziejczyk et al, 2008
dVGlut-Gal4	*w*^{1118}*;dVGlut-Gal4*	*dVGlut*	Collins et al., 2006
ERR^{G4389}	*y[1] w[*]; P{w[+mC]=EP}ERR[G4389]*	*ERR*	Bl# 28467
UAS-ERR^{JF02431}	*y[1] v[1]; P{y[+t7.7] v[+t1.8]=TRiP.JF02431}attP2*	*ERR*	Bl#27085
UAS-CG10672^{HMS00753}	*y[1] sc[*] v[1] sev[21]; P{y[+t7.7] v[+t1.8]=TRiP.HMS00753}attP2*	*CG10672/dPECR*	Bl#32958
CG10672^{KG07864}	*y[1] w[67c23]; P{y[+mDint2] w[BR.E.BR]=SUPor-P}CG10672[KG07864] ry[506]*	*CG10672/dPECR*	Bl#14560
twins$^{CB-5218-3}$	*w*^{1118}*; P{RS3}tws*$^{CB-5218-3}$	*Twins*	Kyoto Stock Center No. 123511
UAS-Ku80^{JF02790}	*y[1] v[1]; P{y[+t7.7] v[+t1.8]=TRiP.JF02790}attP2*	*Ku80*	Bl#27710
Gpdh1$^{CB-6367-3}$	*w*^{1118}*; P{RS3}Gpdh1*$^{CB-6367-3}$	*Gpdh1*	Kyoto Stock Center No. 124074

Flies carrying transposable element insertions marked with *white* were backcrossed to *w*^{1118} (Scholz lab) for at least five generations to isogenize the genetic background.

1. Besson MT, Sinakevitch I, Melon C, Iche-Torres M, Birman S. Involvement of the Drosophila taurine/aspartate transporter dEAAT2 in selective olfactory and gustatory perceptions. *The Journal of comparative neurology* 2011; **519**(14)**:** 2734-2757.
2. Collins CA, Wairkar YP, Johnson SL, DiAntonio A. Highwire restrains synaptic growth by attenuating a MAP kinase signal. *Neuron* 2006; **51**(1)**:** 57-69.
3. Kolodziejczyk A, Sun X, Meinertzhagen IA, Nassel DR. Glutamate, GABA and acetylcholine signaling components in the lamina of the Drosophila visual system. *PloS one* 2008; **3**(5)**:** e2110.
4. Scholz H, Franz M, Heberlein U. The hangover gene defines a stress pathway required for ethanol tolerance development. *Nature* 2005; **436**(7052)**:** 845-847.
5. Torroja L, Chu H, Kotovsky I, White K. Neuronal overexpression of APPL, the Drosophila homologue of the amyloid precursor protein (APP), disrupts axonal transport. *Current biology : CB* 1999; **9**(9)**:** 489-492.

Supplementary Table 2. Primer.

Primer	Sequence	Location	Position*
RplP0-sense	CAGCGTGGAAGGCTCAGTA	*RplP0*	+441
RplP0-anti	CAGGCTGGTACGGATGTTCT	*RplP0*	+617
actin5C-sense	TTAGCTCAGCCTCGCCACTT	*actin5C*	+1168
actin5C-anti	GCAGCAACTTCTTCGTCACA	*actin5C*	+1841
SuTpl-sense	TCCCAGAGCCACCGTTACAC	*Su(Tpl)*	+14774
SuTpl-anti	CTGGTTGCAGGCGTTTAGCGT	*Su(Tpl)*	+14874
Tub-sense	TGTCGCGTGTGAAACACTTC	*alphaTub84B*	+109
Tub-anti	AGCAGTAGAGCTCCCAGCAG	*alphaTub84B*	+689
TrECo fwd	TCTTGCTGACGGAATAGGCT	*CG10672/dPECR*	+878
TrECo rev	GAGTGCGATGAGAAGGTGTG	*CG10672/dPECR*	+658
Hang F1	GAACGGTCGGCGCGACAAAA	*hangover*	+666
Hang R1	CCGATCCTGCGGTGTAACCTGA	*hangover*	+6226
ERR plus	GTGTGAGATTAACAAGCGGAG	*ERR*	+2110
ERR minus	GAGGTGGTGTTGGATTGGTA	*ERR*	+2335
twins sense	TGTTGGTTGGCTTGATGTCG	*Twins*	+10816
twins anti	TCCGTGAAGCAGATACCACT	*Twins*	+10637
Gpdh1 left	GATTATCAACGAGACGCACG	*Gpdh1*	+740
Gpdh1 right	ATCTTGCCCAGTAGCTGTTT	*Gpdh1*	+2489
Ku80 plus	TGAATAAGACAGCCTGCGAG	*Ku80*	+473
Ku80 minus	ATTCGTCCGTGATTTCGTTG	*Ku80*	+679

*Relative to transcription start site.

The control gene used for the qRT-PCRs for *ERR* and tws is *SuTpl*; for *dPECR*, the controls are *actin* and *Gpdh1* and for *Ku80*, the control is *tubulin*.

Transcript levels after ethanol exposure were normalized to the reference level of *Rap2* and to $hang^{AE10}$ mutants with *Su(Tpl)*.

Supplementary Table 3: Putative *Drosophila* orthologue proteins for the proteins associated with SNPs.

The table summarizes the similarity or identity of the amino acids within the region of the protein that share the highest homology to the corresponding human protein.

**SNP**		**Human gene**	**Most similar in *Drosophila***	**In stretch of homology: Identities and Positives**
rs1344694	Intron Variant	**Melanoregulin (MREG)**<br><br>NM_001372190.1<br><br>HGNC:25478	**Karst (kst)**<br><br>CG12008	Identities = 18/56 (32.1%)<br>Positives = 23/56 (41.1%)
rs7590720	Intron Variant	**Peroxisomal trans-2-enoyl-CoA reductase (PECR)***<br><br>**Melanoregulin**<br><br><br>XR_001738847.2<br>HGNC:18281 and HGNC:25478	**dPECR**[a]<br><br>CG10672/ Dehydrogenase/reductase 4 (Dhrs4)	Identities = 92/261 (35.2%)<br>Positives = 136/261 (52.1%)
rs705648	Intron Variant	**Peroxisomal trans-2-enoyl-CoA reductase (PECR)***<br><br>XR_001738847.2<br><br><br>HGNC:18281	**dPECR**[a]<br><br>CG10672/ Dehydrogenase/reductase 4 (Dhrs4)	Identities = 92/261 (35.2%)<br>Positives = 136/261 (52.1%)
rs1614972	Intron Variant	**ADH1C/ADH3**<br><br>NM_000669.5:c.<br><br>HGNC:251	**Formaldehyde dehydrogenase (Fdh)**<br>CG6598	Identities = 199/377 (52.8%)<br>Positives = 265/377 (70.3%)

rs13362120	Intron Variant	**calpastatin (CAST)**<br><br>NM_001750.7:c.<br>HGNC:1515	**N/A**	
rs13160562	Intron Variant	**Endoplasmic reticulum aminopeptidase 1 (ERAP1)**<br><br>NM_016442.4:c.<br><br>HGNC:18173	**CG8773**	Identities = 321/932 (34.4%)<br>Positives = 483/932 (51.8%)
rs1864982	Intron Variant	**Serine/threonine-protein phosphatase 2A 55 kDa regulatory subunit B beta isoform (PPP2R2B)****<br>NM_001271948.1:c.<br><br>HGNC:9305	**Twins (tws)**<br><br>CG6235	Identities = 335/421 (79.6%)<br>Positives = 376/421 (89.3%)
rs6902771	Intron Variant	**Oestrogen receptor 1 (ESR1)**<br><br>NM_000125.3:c.<br><br>HGNC:2467	**Oestrogen-related receptor (ERR)*****<br>CG7404	Identities = 116/328 (35.4%)<br>Positives = 183/328 (55.8%)
rs729302<br><br><br><br><br>rs13273672	Intergenic<br><br><br><br><br>Intron Variant	**Kielin cysteine-rich BMP regulator (KCP)**<br><br>**Interferon regulatory factor 5 (IRF5)**<br><br>HGNC:17585 and HGNC:6120<br>**GATA-binding protein 4 (GATA4)**<br><br>NM_001308093.1:c.<br><br>HGNC:4173	**Crossveinless 2 (cv-2)**<br><br>CG15671<br><br>**N/A**<br>**Pannier (pnr)**<br><br>CG3978	Identities = 211/616 (34.3%)<br>Positives = 287/616 (46.6%)<br><br>Identities = 97/117 (82.9%)<br>Positives = 105/117 (89.7%)
rs1487814	Intron Variant	**LOC102723370**<br><br>XR_001748151.1:n.<br>ncRNA	**N/A**	

rs7138291	Intron Variant	**Centrosomal protein 83 (CEP83)**<br><br>NM_016122.3:c.<br>HGNC:17966	**N/A**	
rs36563	Intergenic	**Pecanex 1 (PCNX1)**<br><br>**Transmembrane protein 183A pseudogene LOC100287335**<br>HGNC:19740<br>HGNC: no protein	**Pecanex (pcx)**<br><br>CG3443<br><br>**N/A**	Identities = 323/534 (60.5%)<br>Positives = 425/534 (79.6%)
rs11640875	Intron Variant	**Cadherin 13 (CDH13)**<br><br>NM_001257.5:c.<br><br>HGNC:1753	**Cadherin-N (Cad-N)**<br><br>CG7100	Identities = 213/719 (29.6%)<br>Positives = 335/719 (46.6%)
rs12388359	Intergenic	**Chloride voltage-gated channel 4 (CLCN4)**<br><br>HGNC: 2022<br><br>**Midline 1 (MID1)**<br><br>HGNC:7095	**Chloride channel-c (ClC-c)**<br><br>CG5284<br><br>**Tripartite motif-containing 9 (Trim9)**<br>CG31721	Identities = 463/759 (61%)<br>Positives = 586/759 (77.2%)<br>Identities = 155/590 (26.3%)<br>Positives = 243/590 (41.2%)
rs9825310	Intergenic	**Glycerol-3-phosphate dehydrogenase 1-like (GPD1)****** <br>HGNC:28956<br><br>**CKLF-like MARVEL transmembrane domain-containing 8 (CMTM8)**<br>HGNC:19179	**Glycerol-3-phosphate dehydrogenase 1 (Gpdh1)**<br>CG9042<br><br>**CG15211**	Identities = 212/349 (60.7%)<br>Positives = 263/349 (75.4%)<br>Identities = 36/143 (25.2%)<br>Positives = 59/143 (41.3%)

[a]The *Drosophila* CG10672/Dhrs4 protein is the most similar to human PECR (35% amino acid identity); thus, this candidate gene is called *dPECR*.

*PECR is expressed in the cytotoxic hydrogen peroxide producing cell organelle peroxisome [1].

**The PPP2R2B protein is a serine/threonine protein phosphatase associated with mitochondria and regulates - oxidative stress-induced apoptosis[2]; [3].

***The *Drosophila* ERR protein is required, together with the hypoxia inducing factor (HIF-1α), for the transcriptional response to hypoxic cellular stress[4].

****The GPD1L protein is a regulator of HIF-1α stability, and the function is linked to the hypoxic cellular stress response via HIF-1α [5].

Ku80 identified as candidate gene for AUD[6] also interacts with HIF-1α [7].

1. Das AK, Uhler MD, Hajra AK. Molecular cloning and expression of mammalian peroxisomal trans-2-enoyl-coenzyme A reductase cDNAs. *J Biol Chem* 2000; **275**(32)**:** 24333-24340.
2. Dagda RK, Zaucha JA, Wadzinski BE, Strack S. A developmentally regulated, neuron-specific splice variant of the variable subunit Bbeta targets protein phosphatase 2A to mitochondria and modulates apoptosis. *J Biol Chem* 2003; **278**(27)**:** 24976-24985.
3. Liu WB, Li Y, Zhang L, Chen HG, Sun S, Liu JP *et al.* Differential expression of the catalytic subunits for PP-1 and PP-2A and the regulatory subunits for PP-2A in mouse eye. *Mol Vis* 2008; **14:** 762-773.
4. Li Y, Padmanabha D, Gentile LB, Dumur CI, Beckstead RB, Baker KD. HIF- and non-HIF-regulated hypoxic responses require the estrogen-related receptor in Drosophila melanogaster. *PLoS Genet* 2013; **9**(1)**:** e1003230.
5. Kelly TJ, Souza AL, Clish CB, Puigserver P. A hypoxia-induced positive feedback loop promotes hypoxia-inducible factor 1alpha stability through miR-210 suppression of glycerol-3-phosphate dehydrogenase 1-like. *Mol Cell Biol* 2011; **31**(13)**:** 2696-2706.
6. Juraeva D, Treutlein J, Scholz H, Frank J, Degenhardt F, Cichon S *et al.* XRCC5 as a risk gene for alcohol dependence: evidence from a genome-wide gene-set-based analysis and follow-up studies in Drosophila and humans. *Neuropsychopharmacology* 2015; **40**(2)**:** 361-371.
7. Liu T, Jin L, Chen M, Zheng Z, Lu W, Fan W *et al.* Ku80 promotes melanoma growth and regulates antitumor effect of melatonin by targeting HIF1-alpha dependent PDK-1 signaling pathway. *Redox Biol* 2019; **25:** 101197.

Supplementary Table 4. Protein-protein interaction.

	Physical interaction	Assay	Reference
**Group 1:**	Pnr-Ada2B	Anti-tag coimmunoprecipitation, peptide mass fingerprinting	Weake et al., 2011
	Ada2B-ERR	Anti-tag coimmunoprecipitation, peptide mass fingerprinting	Weake et al., 2011
	ERR-Sima	Two-hybrid, pull down, autoradiography	Li et al., 2013
	Sima-Hang	Anti-tag coimmunoprecipitation, identification by mass spectrometry	Vinayagam et al., 2016
	TFAM-Ku80	Coaffinity purification coupled to mass spectrometry analysis	Guruharsha et al., 2011
	Hang-TTK	Anti-tag coimmunoprecipitation, peptide mass fingerprinting,	Rhee et al., 2014
	Ku80-TTK	Coaffinity purification coupled to mass spectrometry analysis	Guruharsha et al., 2011

**Group 2:**	InR-Chico	Anti-tag coimmunoprecipitation, identification by mass spectrometry, peptide mass fingerprinting, two hybrid, fluorescence resonance energy transfer	Breitkopf et al., 2016, Vinayagam et al., 2016, Almudi et al., 2013, Friedman et al., 2011, Glatter et al., 2011, Pflieger et al., 2008, Poltilove et al., 2000
	Chico-Twins	Anti-tag coimmunoprecipitation, identification by mass spectrometry	Vinayagam et al., 2016
	S6k-Twins	Anti-tag coimmunoprecipitation, identification by mass spectrometry	Vinayagam et al., 2016

**Group 3:**	Gpdh1-Adh	Coaffinity purification coupled to mass spectrometry analysis	Guruharsha et al., 2011
	Gpdh1-Eno	Coaffinity purification coupled to mass spectrometry analysis	Guruharsha et al., 2011
	Eno-Adh	Coaffinity purification coupled to mass spectrometry analysis	Guruharsha et al., 2011
	Eno-Uch	Coaffinity purification coupled to mass spectrometry analysis	Guruharsha et al., 2011
	Uch-CG6180	Coaffinity purification coupled to mass spectrometry analysis	Guruharsha et al., 2011
	CG6180-dPECR	Coaffinity purification coupled to mass spectrometry analysis	Guruharsha et al., 2011

1. Almudi I, Poernbacher I, Hafen E, Stocker H. The Lnk/SH2B adaptor provides a fail-safe mechanism to establish the Insulin receptor-Chico interaction. *Cell communication and signaling : CCS* 2013; **11**(1)**:** 26.

2. Breitkopf SB, Yang X, Begley MJ, Kulkarni M, Chiu YH, Turke AB *et al.* A Cross-Species Study of PI3K Protein-Protein Interactions Reveals the Direct Interaction of P85 and SHP2. *Scientific reports* 2016; **6:** 20471.

3. Friedman AA, Tucker G, Singh R, Yan D, Vinayagam A, Hu Y *et al.* Proteomic and functional genomic landscape of receptor tyrosine kinase and ras to extracellular signal-regulated kinase signaling. *Science signaling* 2011; **4**(196)**:** rs10.

4. Glatter T, Schittenhelm RB, Rinner O, Roguska K, Wepf A, Junger MA *et al.* Modularity and hormone sensitivity of the Drosophila melanogaster insulin receptor/target of rapamycin interaction proteome. *Molecular systems biology* 2011; **7:** 547.

5. Guruharsha KG, Rual JF, Zhai B, Mintseris J, Vaidya P, Vaidya N *et al.* A protein complex network of Drosophila melanogaster. *Cell* 2011; **147**(3)**:** 690-703.

6. Li Y, Padmanabha D, Gentile LB, Dumur CI, Beckstead RB, Baker KD. HIF- and non-HIF-regulated hypoxic responses require the estrogen-related receptor in Drosophila melanogaster. *PLoS genetics* 2013; **9**(1)**:** e1003230.

7. Pflieger D, Junger MA, Muller M, Rinner O, Lee H, Gehrig PM *et al.* Quantitative proteomic analysis of protein complexes: concurrent identification of interactors and their state of phosphorylation. *Molecular & cellular proteomics : MCP* 2008; **7**(2)**:** 326-346.

8. Poltilove RM, Jacobs AR, Haft CR, Xu P, Taylor SI. Characterization of Drosophila insulin receptor substrate. *The Journal of biological chemistry* 2000; **275**(30)**:** 23346-23354.

9. Rhee DY, Cho DY, Zhai B, Slattery M, Ma L, Mintseris J *et al.* Transcription factor networks in Drosophila melanogaster. *Cell reports* 2014; **8**(6)**:** 2031-2043.

10. Vinayagam A, Kulkarni MM, Sopko R, Sun X, Hu Y, Nand A *et al.* An Integrative Analysis of the InR/PI3K/Akt Network Identifies the Dynamic Response to Insulin Signaling. *Cell reports* 2016; **16**(11)**:** 3062-3074.

11. Weake VM, Dyer JO, Seidel C, Box A, Swanson SK, Peak A *et al.* Post-transcription initiation function of the ubiquitous SAGA complex in tissue-specific gene activation. *Genes & development* 2011; **25**(14)**:** 1499-1509.

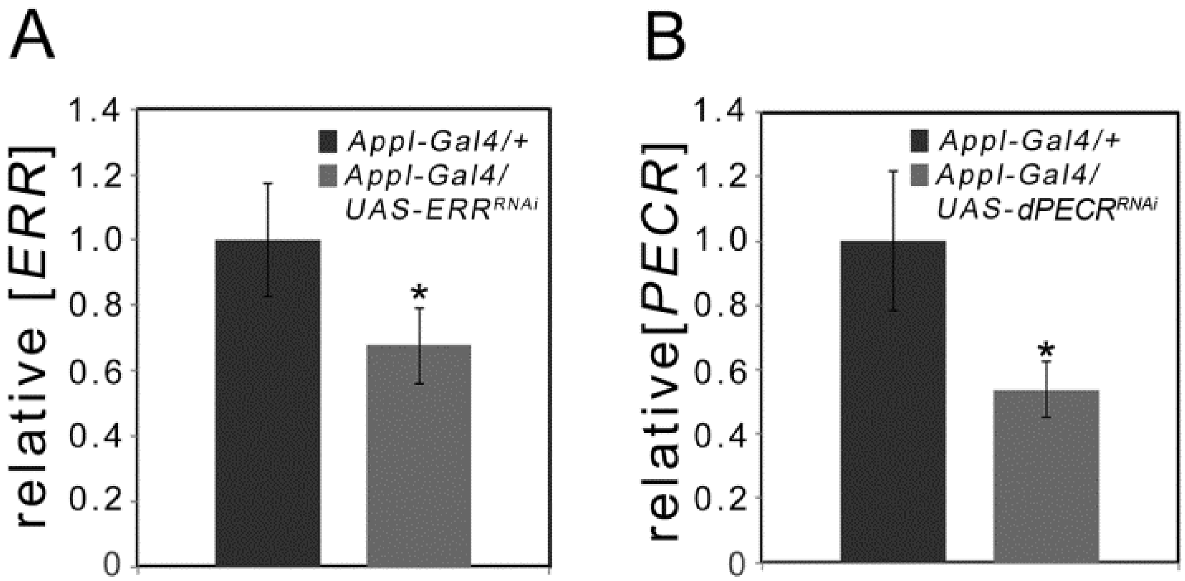

Supplementary Fig. 1. Validation of RNAi transgenes for *ERR* and *PECR* using qRT-PCR.

Transcription levels of the candidate genes were determined in cDNA from adult fly heads using qRT-PCR. (a) Expression of the $UAS\text{-}ERR^{JF02431}$ RNAi transgene under the control of the *Appl*-Gal4 driver resulted in a significant reduction in *ERR* levels (0.68 ± 0.057; $P < 0.05$). (b) The expression of the $UAS\text{-}PECR^{HMS0075}$ RNAi transgene under the control of the *Appl*-Gal4 driver resulted in a significant reduction in PECR levels (0.54 ± 0.04; $P < 0.05$). The levels of the candidate gene transcripts were compared to heterozygous *Appl*-Gal4 flies. Primers for tubulin were used as controls. For each experiment, N = 4 with 3 technical replicates for each. The data are presented as the means and error bars are STDEVs. Significant differences between two groups were determined using Student's *t*-tests with * $P < 0.05$.
